# Long-term pollen season trends of *Fraxinus* (ash), *Quercus* (oak) and *Ambrosia artemisiifolia* (ragweed) as indicators of anthropogenic climate change impact

**DOI:** 10.1007/s11356-024-34027-w

**Published:** 2024-06-19

**Authors:** Jana Ščevková, Natália Štefániková, Jozef Dušička, Janka Lafférsová, Eva Zahradníková

**Affiliations:** 1 Faculty of Natural Sciences, Department of Botany, Comenius University in Bratislava, Révová 39, 811 02 Bratislava, Slovakia; 2 Department of Environmental Biology, Public Health Office, Banská Bystrica, Slovakia

**Keywords:** Pollen allergens, Meteorological trends, *Quercus*, *Fraxinus*, *Ambrosia*

## Abstract

The ongoing climatic change, together with atmospheric pollution, influences the timing, duration and intensity of pollen seasons of some allergenic plant taxa. To study these influences, we correlated the trends in the pollen season characteristics of both woody (*Fraxinus*, *Quercus*) and herbaceous (*Ambrosia*) taxa from two pollen monitoring stations in Slovakia with the trends in meteorological factors and air pollutants during the last two decades. In woody species, the increased temperature during the formation of flower buds in summer and autumn led to an earlier onset and intensification of next year’s pollen season, especially in *Quercus*. The increase of relative air humidity and precipitation during this time also had a positive influence on the intensity of the pollen season of trees. The pollen season of the invasive herbaceous species *Ambrosia artemisiifolia* was prolonged by increased temperature and humidity during the summer and autumn of the same year, which extended the blooming period and delayed the end of the pollen season. From the studied air pollutants, only three were found to correlate with the intensity of the pollen season of the studied taxa, CO − positively and SO_2_ and NO_2_ − negatively. It is important to study these long-term trends since they not only give us valuable insight into the response of plants to changing conditions but also enable the prognosis of the exacerbations of pollen-related allergenic diseases.

**Supplementary Information:**

The online version contains supplementary material available at 10.1007/s11356-024-34027-w.

## Introduction

Anthropogenic climate change, manifested mainly by global warming, leads to spatiotemporal changes in the intensity, duration and/or timing of the pollen season of some allergenic plant taxa (Bogawski et al. 2014; Lind et al. 2016; Ščevková et al. 2021; Rojo et al. 2021; Anderegg et al. 2021). These changes are associated with detrimental effects on the well-being of pollen allergy sufferers, mainly due to more intense and prolonged exposure to causative pollen allergens or exposure to new types of aeroallergens resulting from the climate-induced spread of invasive species to new areas (Tu et al. 2021). The impact of climate change on the pollen season timing is evidenced by an earlier start of the pollination period, especially for spring pollinating arboreal species (Gehrig and Clot 2021; Levetin 2021) and a later end of the flowering period, especially for summer‒autumn pollinating herbaceous species (Bogawski et al. 2014; Lind et al. 2016).

To capture the temporal trends, we selected taxa whose pollen seasons cover the beginning and end of the vegetation period, are abundant in the study areas and have the potential to trigger pollen allergies. Two arboreal pollen genera—*Fraxinus* (ash) and *Quercus* (oak)—and one herbaceous species—*Ambrosia artemisiifolia* (ragweed)—fulfil these conditions. In Slovakia, the pollen season of the chosen trees starts at the end of March and lasts until the beginning of May for ash and from the end of April to the beginning of June for oak (Jurko 1990), while the pollination period of ragweed is seen between the beginning of July and the end of October (Hrabovský et al. 2016). According to skin prick tests, their pollen grains are among the strong pollen allergens in Slovakia (Hrubiško 1998).

Several species of *Fraxinus* and *Quercus* are naturally present in the forests surrounding the studied areas—Bratislava and Banská Bystrica in Slovakia (Petrášová and Jarolímek 2012)—and also planted along roadsides and riverbanks, in parks and other green areas of cities (Reháčková and Pauditšová, 2004). Ragweed is an annual herbaceous plant species native to North America. In Europe, it is an invasive and alien species (Hamaoui-Laguel et al. 2015) that poses a threat to public respiratory health due to the high allergic potential of its pollen (Wopfner et al. 2005). The Pannonian Plain, which includes a part of Slovakia, is considered the most ragweed-infested area in Europe (Skjøth et al. 2019). Predominantly, its prevalence within Slovakia manifests in the southwestern and south-eastern territories, exhibiting a progressive northward expansion facilitated by transportation networks such as roads and highways (Hrabovský et al. 2016).

A positive feedback loop exists between anthropogenic climate change and chemical atmospheric pollution. There has been increasing discussion about the role of air pollutants in the rising prevalence of pollen-related allergic diseases, such as rhinitis or asthma, especially in urban areas (Takizawa 2011; D’Amato et al. 2015; Berger et al. 2020). Indeed, a higher concentration of CO_2_ has been shown to result in increased ragweed and oak pollen production and raising the allergen concentration of the pollen (Rogers et al. 2006; El Kelish et al. 2014; Ziska 2021). Other atmospheric pollutants like O_3_, NO_2_, CO, SO_2_ and PM particles act as plant stressors. They can alter the severity of pollen seasons by increasing the allergenic potential of pollen grains. This happens because plants exposed to higher levels of environmental stress tend to produce more defence-related allergenic proteins (Gilles et al. 2018; Rauer et al. 2021). It has been shown that these levels of stress also influence pollen production either positively (Zhao et al. 2017) or negatively (Jochner et al. 2013).

Considering this effect, it is estimated that airborne pollen concentration of allergenic plant taxa and their allergenicity will continue to rise in the future (El Kelish et al. 2014; Hamaoui-Laguel et al. 2015). Combined with the predicted spread of invasive plant species, such as ragweed (Cunze et al. 2013), a considerable impact on the sensitised population can be expected. A few studies in Europe have addressed this problem so far, primarily from the perspective of global warming (Bogawski et al. 2014; Hamaoui-Laguel et al. 2015; Rodinkova et al. 2018; Ruiz-Valenzuela and Aguilera 2018; Glick et al. 2021; Gehrig and Clot 2021; Ščevková et al. 2021; Adams-Groom et al. 2022). However, studies simultaneously considering air pollutants and meteorological factors affecting the long-term pollen season trends are scarce (Oduber et al. 2019).

In this study, we investigated the trends in the intensity, timing and duration of *Fraxinus*, *Quercus* and *Ambrosia artemisiifolia* pollen seasons over the last two decades at two pollen monitoring stations in Slovakia and the relationship between these trends and the meteorological and air pollution trends over time.

## Materials and methods

### Pollen sampling sites

**Fig. 1:**
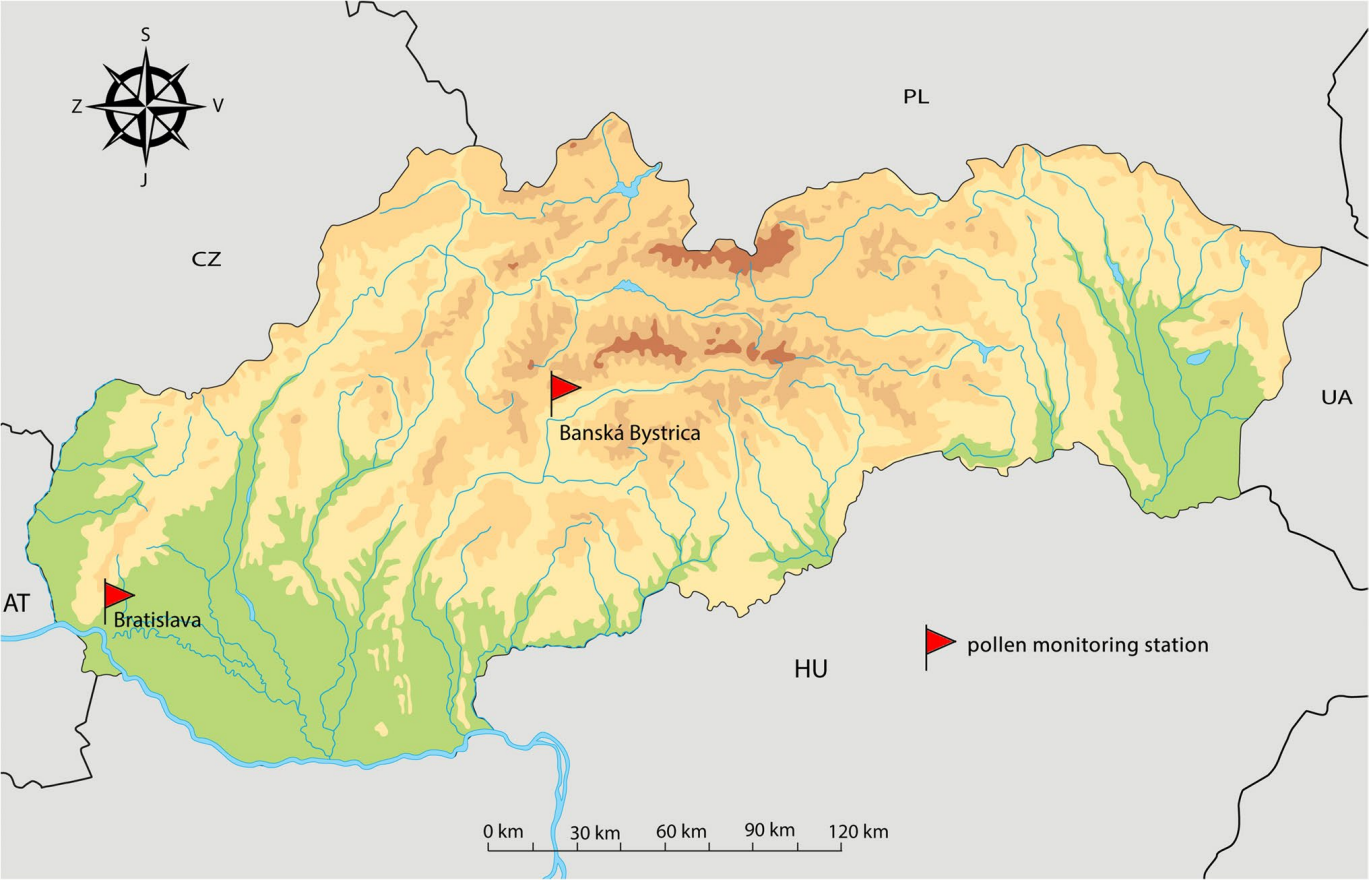
Location of the pollen monitoring sites in Slovakia

Two sites, Bratislava (48.14944° N, 17.07333° E) and Banská Bystrica (48.74201° N, 19.16531° E), hereafter B. Bystrica, situated in the southwestern and central part of Slovakia, respectively (Fig. 1), had sufficient data sets for the trend analysis. Bratislava, with an altitude ranging from 126 to 514 m a. s. l., lies at the boundary of the Malé Karpaty Mts. and Podunajská nížina Lowland and is surrounded by agricultural fields, vineyards and deciduous forests. B. Bystrica is situated in a long and wide valley encircled by mountain chains of the Nízke Tatry, Veľká Fatra and Kremnica Mts., at an altitude of 362 m a. s. l. The city is predominantly surrounded by woodlands. The climate on the sites is continental, with long-lasting winters and hot (Bratislava) and mild (B. Bystrica) summers. In Bratislava, the mean annual temperature is 11.2 °C and total annual precipitation is 748 mm, while in B. Bystrica, it is 9.7 °C and 713 mm respectively (2000–2021 average, Slovak Hydrometeorological Institute – SHMÚ). North-western winds prevail in both sites (SHMÚ).

### Aerobiological data

The period of the study corresponds to pollen seasons 2002 − 2022 in Bratislava and 2002 − 2021 in B. Bystrica, from February to November. Samples were obtained using Hirst-type volumetric pollen traps (Hirst 1952) placed on the rooftops at 18 (Bratislava) and 10 (B. Bystrica) m a. g. l. and analysed according to standard aerobiological methods (Galán et al. 2007). Daily means of pollen concentration, expressed as a number of pollen grains per cubic metre of air (pollen/m^3^), were calculated.

The main pollen season (MPS) was set as the period between the first and the last day of the pollen season when pollen concentration reached a daily average equal to or greater than 10 pollen/m^3^ (Peel et al. 2014).

To evaluate the impact on human health, we noted the first day when the mean daily pollen concentration reached the threshold value of 50 pollen/m^3^ for *Fraxinus* and *Quercus*, a moderate tree pollen concentration according to Frenz (2021) and 20 pollen/m^3^ for *Ambrosia* (Stępalska et al. 2008) provoking the first symptoms in sensitised patients. Days when the pollen concentration reached a daily average equal to or greater than the threshold value were defined as high days (HD) and the first day when the threshold value was reached was a first high day (FHD).

### Data analysis

#### Pollen, meteorological and air pollution trends

Statistically significant *Fraxinus*, *Quercus* and *Ambrosia* pollen trends, as well as meteorological and air pollution trends, were investigated using a non-parametric Mann–Kendall trend test for two sites, using annual means for 21 years (Bratislava) and 20 years (B. Bystrica). The test reveals monotonic trends in a time series by computing Kendall's tau correlation coefficients between time and each considered variable. A positive or negative tau value denotes an increasing or decreasing trend within a data series. In cases where a significant trend is detected, a simple straight-line regression model is employed to model it. The Theil-Sen non-parametric method was used to determine the true slope of any linear trend (Theil 1950).

Seven pollen trends were investigated: (1) the start and (2) the end date of MPS, expressed as the day of the year (DOY) counted from January 1st, (3) the Seasonal Pollen Integral (SPIn), obtained by summing the average daily pollen concentrations over the MPS (pollen*day/m^3^), (4) the peak value (pollen/m^3^) ‒ the highest daily pollen concentration of the MPS, (5) the duration (number of days) of the MPS, (6) the number of HD and (7) FHD (DOY).

Meteorological and air pollution trends were analysed for each month as well as for all intervals of two or more months during pre-season (from the formation of flower buds until the start of MPS) and in-season (MPS, rounded up/down to full months). This interval lasts from May of the preceding year to April for *Fraxinus*, from June of the preceding year to May for *Quercus* and from April to October for *Ambrosia*. The following meteorological data were included in the trend analysis: mean (T_mean_), maximum (T_max_) and minimum (T_min_) daily average temperature (°C), relative air humidity (%), precipitation (mm) and sunshine (h). Concerning air pollutants, particulate matter ≤ 10 μm (PM_10_), ozone (O_3_), carbon monoxide (CO), nitrogen dioxide (NO_2_) and sulphur dioxide (SO_2_), all expressed in µg/m^3^, were included in the trend analysis. All meteorological and air pollution data were provided by the Slovak Hydrometeorology Institute. In Bratislava, the distance between the meteorological and the pollen monitoring station is about 0.5 km and in B. Bystrica it is about 3.7 km. Four hundred sixty-eight meteorological and 390 air pollution trends altogether were investigated for their statistical significance using above-mentioned Kendall’s tau correlation analysis.

#### Influences of meteorological and air pollution parameters on pollen trends

Spearman ‘s correlation coefficients were used to establish the relationship between the seven above-mentioned pollen season–related characteristics and significant meteorological trends for both analysed sites. To analyse the impact of air pollutants on the intensity of pollen seasons (SPIn, peak pollen values and HD) of the analysed taxa, Spearman ‘s correlation coefficients were calculated between the air pollutants with a significant change in concentration during the last 20 years and these characteristics. All data analyses were performed in Statistica 12.

## Results

### Meteorological and air pollution trends

Significant meteorological and air pollution trends were identified in both study areas: three meteorological and four decreasing air pollution trends were revealed in Bratislava and four meteorological and four air pollution trends (two rising and two decreasing) in B. Bystrica (Table 1). A rise in air temperature and either precipitation or relative air humidity have been observed on both sites over the studied period. On the other hand, the air pollution trends tended to be negative, except for rising CO concentration in B. Bystrica.

**Table 1:** Significant meteorological and air pollution trends in Bratislava (2002 − 2022) and B. Bystrica (2002 − 2021) calculated from homogeneous series: Mann–Kendall trend test (test *tau* column), Sen’s slope estimation (*Q* column)

Site	Environmental series	*tau*	*Q*
Bratislava	T_mean_ June − October	0.486**	0.06
	T_min_ June − October	0.513**	0.07
	P September − November	0.419**	2.47
	SO_2_ March − May	− 0.716***	− 0.51
	SO_2_ June − October	− 0.558***	− 0.4
	NO_2_ March − April	− 0.562***	− 1.07
	NO_2_ June − August	− 0.543***	− 0.56
B. Bystrica	T_mean_ June − October	0.474*	0.06
	T_min_ October − December	0.44**	0.12
	T_max_ June − December	0.379*	0.09
	RH October − December	0.484**	0.33
	CO March − May	0.516***	18.17
	CO June − September	0.432**	14.94
	SO_2_ April	− 0.379*	− 0.24
	SO_2_ October − December	− 0.568***	− 0.41

^*^p < 0.05; **p < 0.01; ***p < 0.001

*T*_*mean*_, mean air temperature; *T*_*min*_, minimum air temperature; *T*_*max*_, maximum air temperature;* P*, precipitation; *RH*, relative humidity; *CO*, carbon monoxide; *NO*_*2*_, nitrogen dioxide; *SO*_*2*_, sulphur dioxide

### Pollen trends

#### Timing of pollen season

On average, the start of *Quercus* and *Ambrosia* pollen seasons commenced concurrently in both studied areas, occurring in the first fortnight of April and August, respectively. However, the onset of the *Fraxinus* pollen season was delayed by 13 days in B. Bystrica compared to Bratislava where it started in the first fortnight of March (Table 2). The FHD of *Quercus* coincided at both sites, while in B. Bystrica, a shift towards later FHD was observed for *Fraxinus* (18 days) and *Ambrosia* (23 days).

**Table 2:** Descriptive statistics of *Fraxinus*, *Quercus* and *Ambrosia* pollen season parameters in Bratislava (2002 − 2022) and B. Bystrica (2002 − 2021)

		Fraxinus				Quercus				Ambrosia			
Site	Pollen season parameter	Mean	Max	Min	SD	Mean	Max	Min	SD	Mean	Max	Min	SD
Bratislava	Start date (DOY)	73	98	47	15.8	101	118	77	11.9	222	229	200	6.3
	End date (DOY)	112	124	96	8	129	144	103	9.1	269	286	254	7
	Duration (days)	39	70	7	15.8	29	59	9	13.9	48	87	33	10.8
	Peak value (pollen/m^3^)	375	1448	22	430	234	1158	25	258.8	218	414	37	107.9
	High days (number)	11	36	0	10.1	7	22	0	6.5	23	41	8	7.1
	First high day (DOY)	82	110	54	15.8	110	121	78	10.8	227	234	210	5
	SPIn (pollen*day/m^3^)	2250	9143	108	2348.5	1426	6808	133	1551.3	1779	2883	659	549.3
B. Bystrica	Start date (DOY)	87	113	70	10.1	103	120	70	14.5	220	236	201	9.5
	End date (DOY)	133	160	120	10.8	142	160	129	8.4	266	301	237	14.4
	Duration (days)	47	71	21	14.3	40	86	17	18.4	47	82	15	17.8
	Peak value (pollen/m^3^)	148	600	22	164.6	189	538	22	149.7	186	536	18	127
	High days (number)	9	25	0	8.6	9	35	0	8.2	15	34	3	8.3
	First high day (DOY)	100	122	78	13.7	111	137	70	15.7	250	277	228	13.8
	SPIn (pollen*day/m^3^)	1154	4357	234	1095.7	1262	4040	197	1072.2	1028	2584	151	733.6

*DOY*, day of the year from 1 January

The years with the most significant deviations from the average in the timing of pollen seasons were 2020 for tree species in both sites and 2018 and 2010 for *Ambrosia* in Bratislava and B. Bystrica, respectively. In 2020, the onset of pollen seasons for *Fraxinus* and *Quercus* occurred 17 to 33 days earlier than the long-term average starting date and the corresponding FHD for these taxa were observed 21 to 41 days earlier than the long-term average date of FHD (Tables S1 and S2). In 2018, *Ambrosia* pollen season in Bratislava started 22 days earlier and FHD was reached 17 days earlier than the long-term average (Table S3). In B. Bystrica, the *Ambrosia* pollen season started 18 days earlier and the FHD was advanced 18 days in 2010.

The long-term trend for both sites (Table 3) was an earlier start and later end of the pollen seasons of all three analysed taxa; however, the trends for the start day of the pollen season have greater statistical significance in most cases. Significant trends for the end of the pollen season were only found for *Ambrosia*. A trend of earlier occurrence of FHD was noted for *Fraxinus* and *Quercus*, while an opposite shift was observed for *Ambrosia*, although statistically significant only in B. Bystrica.

**Table 3:** The Mann–Kendall trend test (test *tau* column) and Sen’s slope estimate (*Q* column) of *Fraxinus*, *Quercus* and *Ambrosia* pollen season parameters in Bratislava (2002 − 2022) and B. Bystrica (2002 − 2021)

Site	Pollen season parameter	*Fraxinus*		*Quercus*		*Ambrosia*	
		*tau*	*Q*	*tau*	*Q*	*tau*	*Q*
Bratislava	Start date	− 0.369*	− 1.5	− 0.433**	− 1.23	− 0.316*	− 0.33
	End date	− 0.16	− 0.33	0.174	0.38	0.301^+^	0.5
	Duration	0.239	1.03	0.413**	1	0.564***	0.88
	Peak value	0.219	11.73	0.41**	14.4	0.167	5.12
	High days	0.239	0.58	0.349*	0.5	0.155	0
	First high day	− 0.306^+^	− 1.42	− 0.294^+^	− 0.71	− 0.035	0.21
	SPIn	0.362*	135.63	0.448**	104	0.19	25.6
B. Bystrica	Start date	− 0.209	− 0.42	− 0.557***	− 1.62	− 0.323*	− 0.71
	End date	0.186	0.41	0.264	0.67	0.468**	1.24
	Duration	0.24	1	0.646***	2.16	0.546**	2.09
	Peak value	0.263	7.78	0.354*	16	0.221	10.7
	High days	0.443**	0.94	0.488**	0.75	0.349*	0.62
	First high day	− 0.309^+^	− 1.28	− 0.553***	− 1.14	0.462**	1.64
	SPIn	0.347*	66.7	0.421**	43.6	0.263	37.7

^+^*p* < 0.1; *p < 0.05; **p < 0.01; ***p < 0.001

Based on the results of correlation analysis between the pollen season parameters and environmental trends, shown in Table 4, we found that an increase in temperature during pre-season caused an earlier onset of the pollen season for trees, significant for *Fraxinus* in Bratislava and *Quercus* in B. Bystrica. The significant delay in the *Ambrosia* pollen season end date is matched by rising air temperature during its blooming period, more significantly in B. Bystrica. The earlier FHD of trees was negatively correlated with rising air temperature and with relative air humidity or precipitation during pre-season. On the other hand, the later FHD of *Ambrosia* in B. Bystrica was positively correlated with the rising temperatures in summer and autumn.

**Table 4:** Spearman’s correlation coefficients between the significant trends in pollen season parameters of *Fraxinus*, *Quercus* and *Ambrosia* and meteorological series, recorded in Bratislava and B. Bystrica over the last two decades

Site	Taxa	Meteorological series	Start date	End date	Duration	Peak	HD	FHD	SPIn
Bratislava	*Fraxinus*	T_mean_ June − October^a^	− 0.424^+^	-	-	-	-	− 0.439^+^	0.418^+^
		T_min_ June − October^a^	− 0.396^+^	-	-	-	-	− 0.301	0.41^+^
		P September − November^a^	− 0.556*	-	-	-	-	− 0.53*	0.465*
	*Quercus*	T_mean_ June − October^a^	− 0.363	-	0.388^+^	0.245	0.213	− 0.235	0.313
		T_min_ June − October^a^	− 0.368	-	0.277	0.288	0.202	− 0.203	0.305
		P September − November^a^	− 0.256	-	0.208	0.492*	0.445*	− 0.21	0.438^+^
	*Ambrosia*	T_mean_ June − October^b^	− 0.136	0.469*	0.391^+^	-	-	-	-
		T_min_ June − October^b^	− 0.212	0.474*	0.483*	-	-	-	-
		P September − November^b^	− 0.117	0.181	0.294	-	-	-	-
B. Bystrica	*Fraxinus*	T_mean_ June − October^a^	-	-	-	-	0.291	− 0.524*	0.332
		T_min_ October − December^a^	-	-	-	-	0.491*	− 0.372	0.38
		T_max_ June − December^a^	-	-	-	-	0.509*	− 0.457*	0.547*
		RH October − December^a^	-	-	-	-	0.346	− 0.176	0.368
	*Quercus*	T_mean_ June − October^a^	− 0.47*	-	0.276	0.122	0.238	− 0.504*	0.182
		T_min_ October − December^a^	− 0.434^+^	-	0.564*	0.266	0.329	− 0.522*	0.411
		T_max_ June − December^a^	− 0.442^+^	-	0.308	0.326	0.367	− 0.577**	0.367
		RH October − December^a^	− 0.263	-	0.397^+^	0.587**	0.602**	− 0.467*	0.561*
	*Ambrosia*	T_mean_ June − October^b^	− 0.154	0.921***	0.776***	-	0.524*	0.597**	-
		T_min_ October − December^b^	− 0.15	0.539*	0.498*	-	0.441^+^	0.306	-
		T_max_ June − December^b^	− 0.345	0.779***	0.759***	-	0.619**	0.402^+^	-
		RH October − December^b^	− 0.273	0.57**	0.554*	-	0.502*	0.326	-

^+^*p* < 0.1; *p < 0.05; **p < 0.01; ***p < 0.001

^a^Pre-season; ^b^in-season; *T*_*mean*_, mean air temperature; *T*_*min*_, minimum air temperature; *T*_*max*_, maximum air temperature;* P*, precipitation; *RH*, relative humidity

#### Duration of pollen season

During the study period, the *Ambrosia* pollen season lasted, on average, 48 days in Bratislava and 47 days in B. Bystrica (Table 2). The pollen season of *Fraxinus* and *Quercus* in Bratislava lasted, on average, 39 and 29 days, respectively, while in B. Bystrica it was 8 and 11 days longer.

The greatest extension of the pollen season compared to the long-term average for *Ambrosia* was noted in Bratislava in 2018 (39 days longer) (Table S1). For both *Fraxinus* and *Quercus*, the longest seasons were noted in B. Bystrica in 2020: 24 and 46 days longer.

In both sites, we noted a trend of prolongation of the *Quercus* and *Ambrosia* pollen seasons (Table 3). In Bratislava, significant correlations were noted between the duration of the *Ambrosia* pollen season and summer − autumn temperature (Table 4). In B. Bystrica, the duration of the pollen season of both taxa was positively related to air temperature and relative humidity.

#### Intensity of pollen season

The intensity of pollen season is characterised by SPIn, peak value and HD (Table 2). The long-term average value of SPIn was 1426 (*Quercus*), 1779 (*Ambrosia*) and 2250 pollen*day/m^3^ (*Fraxinus*) in Bratislava, while in B. Bystrica, it was 12%, 42% and 49% lower, respectively.

At both sites, we noted trends towards the intensifying of tree pollen seasons; however, all three parameters were statistically significant only in *Quercus* (Table 3), correlated with increased relative humidity and/or precipitation pre-season (Table 4). The significant trends of *Fraxinus* pollen season intensity were associated mostly with increased pre-season temperature. The trend towards intensifying of *Ambrosia* pollen seasons was not significant in Bratislava, while in B. Bystrica, we found a significant correlation between HD and in-season air temperature and relative humidity.

From the studied air pollutants, only three (SO_2_, NO_2_ and CO) were found to have a significant trend that could be correlated with at least one of three pollen season intensity characteristics, SPIn, HD and peak value (Table 5). In tree species, SPIn was negatively correlated with the concentration of SO_2_ both in-season and pre-season (although not significantly for *Fraxinus* in Bratislava in-season), while the correlation with HD was only significant in B. Bystrica, similar to HD of *Ambrosia*. The concentration of SO_2_ was also negatively associated with the peak pollen value for *Quercus*. The concentration of NO_2_ was also negatively associated with the pollen season intensity, particularly significant for *Quercus* in Bratislava. The only air pollutant with rising trends was CO in B. Bystrica, coinciding with the intensity of the pollen season of all three analysed taxa, more pronounced for trees.

**Table 5:** Spearman’s correlation coefficients between the significant trends in the intensity of the *Fraxinus*, *Quercus* and *Ambrosia* pollen season (expressed as SPIn, HD and peak value) and air pollution series, recorded in Bratislava and B. Bystrica over the last two decades

Site	Taxa	Air pollution series	Peak	HD	SPIn
Bratislava	*Fraxinus*	SO_2_ March − May^b^	-	-	− 0.342
		SO_2_ June − October^a^	-	-	− 0.559*
		NO_2_ March − April^b^	-	-	− 0.16
		NO_2_ June − August^a^	-	-	− 0.224
	*Quercus*	SO_2_ March − May^b^	− 0.339	− 0.321	− 0.426^+^
		SO_2_ June − October^a^	− 0.38^+^	− 0.378	− 0.51*
		NO_2_ March − April^b^	− 0.37^+^	− 0.448*	− 0.466*
		NO_2_ June − August^a^	− 0.182	− 0.221	− 0.361
	*Ambrosia*	SO_2_ March − May^a^	-	-	-
		SO_2_ June − October^b^	-	-	-
		NO_2_ March − April^a^	-	-	-
		NO_2_ June − August^b^	-	-	-
B. Bystrica	*Fraxinus*	SO_2_ April^b^	-	− 0.544*	− 0.623**
		SO_2_ October − December^a^	-	− 0.416^+^	− 0.482*
		CO March − May^b^	-	0.577**	0.567**
		CO June − September^a^	-	0.344	0.428^+^
	*Quercus*	SO_2_ April^b^	− 0.581**	− 0.597**	− 0.583**
		SO_2_ October − December^a^	− 0.337	− 0.509*	− 0.405^+^
		CO March − May^b^	0.637**	0.756***	0.71***
		CO June − September^a^	0.402^+^	0.566*	0.477*
	*Ambrosia*	SO_2_ April^a^	-	− 0.485*	-
		SO_2_ October − December^b^	-	− 0.49*	-
		CO March − May^a^	-	0.429^+^	-
		CO June − September^b^	-	0.509*	-

^+^*p* < 0.1; *p < 0.05; **p < 0.01; ***p < 0.001

^a^Pre-season; ^b^in-season; *CO*, carbon monoxide; *NO*_*2*_, nitrogen dioxide; *SO*_*2*_, sulphur dioxide

## Discussion

Plant physiology, allergenicity of pollen grains and pollen season characteristics can be modified by the changing climate (Gehrig and Clot 2021) and the related changes in air pollution (D’Amato et al. 2020). In the present study, we focused on the changes in pollen seasons of common Slovak tree species *Fraxinus* and *Quercus*, and a highly allergenic invasive species *Ambrosia artemisiifolia* (Hrabovský et al. 2016). We studied seven pollen season characteristics in two areas and found a significant change in three to six of them per taxon, correlated with rising air temperature, precipitation, relative air humidity and changes in air pollutant concentration.

### Timing of pollen seasons

We noted a trend towards an earlier pollen season start date and later end date of most analysed taxa, although not always statistically significant. De Weger et al. (2021) found that prolonging the study period from short to long term led to the revelation of novel, previously undetected significant trends. Therefore, we anticipate that some of the current weaker or non-significant pollen trends will gain statistical significance in future long-term studies.

In a comprehensive literature review conducted by Schramm et al. (2021), the investigation of long-term changes in pollen seasons revealed a consistent association between elevated temperatures and an earlier onset of pollen seasons. Similarly, the results of our study indicate a correlation between pre-season temperature which accelerates flowering, and the earlier start of pollen season in tree species. This finding is further supported by the fact that in 2019, when Europe was hit by a heatwave manifested by an increase in the mean June − December daily average temperature by 1.5 °C in Bratislava and 1.1 °C in B. Bystrica compared to the long-term average (2002 − 2021, SHMÚ), the onset of ash and oak pollen seasons was exceptionally early (up to 33 days) in 2020, compared to the average. In the invasive species *Ambrosia artemisiifolia* which reacts favourably to a warmer climate, the earlier onset of flowering is related to precipitation pre-season, in the period from sprouting to pollen maturation. This is in line with the study of Zhao et al. (2023), who found that water availability had a significant positive effect on the establishment rate of ragweed. We found that the highest rainfall total since 2002 (SHMÚ): 493.2 mm in 2018 (Bratislava) and 600.7 mm in 2010 (B. Bystrica) led to a significant shift in the onset of the *Ambrosia* pollen season (up to 22 days earlier).

In contrast to the start of the pollen season, we observed a positive correlation between FHD and summer–autumn temperature in B. Bystrica. However, the start of the pollen season and FHD do not necessarily move in the same direction. In the case of ragweed, in addition to an earlier start, we observed a later end and an overall extension of the pollen season, with no change in pollen intensity. This suggests that the earlier start of the pollen season, which was not driven by temperature, was not associated with intense pollination but with a gradual release of pollen. As ragweed starts flowering earlier, when temperatures are still lower, airborne pollen concentrations will also be lower and only reach FHD due to gradually increasing temperatures.

Similar to Lind et al. (2016), who demonstrated a trend towards a significantly later end of the pollen season for herbaceous taxa in a 40-year study conducted in Stockholm, we have also identified a shift towards a delayed end date of the ragweed pollen season in Slovakia. This shift has been attributed to rising summer‒autumn temperatures, likely influenced by the delayed onset of first autumn frost as suggested by Ziska et al. (2019). Decreasing temperature at the end of the vegetation period is a more significant limiting factor in B. Bystrica than in Bratislava since it lies in a colder region of Slovakia. Furthermore, our study revealed a positive correlation between the end date of the ragweed pollen season and air humidity in autumn. We hypothesise that in a temperate continental climate, the combination of warm and humid weather conditions towards the end of summer and autumn enables an extended blooming period for ragweed.

### Duration of pollen seasons

A consistent and prevailing trend towards extended pollen seasons has been observed in recent studies encompassing multiple pollen stations across different geographical regions and analysing various types of airborne pollen (Lind et al. 2016; Ziska et al. 2019; Rojo et al. 2021; Anderegg et al. 2021). The present study aligns with these findings and identifies the duration of the ragweed and oak pollen season as the most pronounced pollen trend in both stations. The prolonging of *Quercus* pollen season is mostly related to its earlier beginning, while in *Ambrosia* pollen season, we observe a shift towards a later end. This phenomenon can be attributed to the influence of elevated temperature during summer − autumn periods (pre-season for oak and in-season for ragweed), as the rising temperature has been shown to positively affect plant and pollen productivity, especially in urban areas (Schramm et al. 2021; D’Amato et al. 2023).

### The intensity of pollen seasons

The intensification of tree pollen seasons was reported by several long-term aeropalynological studies (Ruiz-Valenzuela and Aguilera 2018; Oduber et al. 2019; Glick et al. 2021). During the course of our study, the intensity of tree pollen seasons, expressed as SPIn, peak value and HD, increased at both localities, although all three characteristics were significant only for oak. Our results agree with the conclusions of Adams-Groom et al. (2022) and Ščevková et al. (2023) that warmer and humid weather in the latter half of the year before pollination stimulates pollen production in woody plants, increasing the intensity of their pollen season.

The intensity of ragweed pollen season has been predicted to rise under changing climate conditions (Rogers et al. 2006; Ziska et al. 2019; Anderegg et al. 2021). However, we did not note a significant rising trend in its parameters, except HD in B. Bystrica. Other authors have even observed a decrease in the intensity of ragweed pollen season (Damialis et al. 2007; Zhang et al. 2015; Levetin 2021). We hypothesise that the absence of significant trends in the Bratislava area is caused by the intense urbanisation in the last decades since it became the capital of Slovakia, reducing localities suitable for the growth of synanthropic plants like *Ambrosia*. In B. Bystrica, we found the rising temperature and relative humidity during summer and autumn positively influenced ragweed HD. This is in line with Cheng et al. (2023), who found that rising temperature promotes the growth of ragweed plants and increases the size of their male inflorescence within a controlled experiment, and Levetin (2021) who found a correlation between autumn humidity and peak value. Since we only observed a significant trend in HD and not in SPIn and peak value, we assume a possible connection with long-distance *Ambrosia* pollen transport from the more infested areas situated in Hungary, as it is a common phenomenon in Europe (Makra et al. 2016; Grewling et al. 2019; Stępalska et al. 2020).

Air pollution levels have improved in Europe over the past 20 years (Chen et al. 2024) due to the environmental and climate policies (e.g. eliminating the burning of coal and fossil fuels, new technologies in the automotive industry) that have been implemented, which was confirmed by our results, as we observed a decrease in the concentration of all analysed pollutants, except CO in B. Bystrica. The only pollutant that was found to intensify the pollen season of the studied taxa was CO, but it is difficult to isolate the influence of this parameter from meteorological factors which significantly impact the concentration of chemical pollutants in the air (Ulutaş et al. 2021). Similar to our results, Ziska and Caulfield (2000) found a positive influence of carbon oxides, together with temperature and sunshine, on plant growth. Other pollutants, especially SO_2_ and NO_2_, can lower the intensity of the pollen season as they can restrict pollen production (Jochner et al. 2013). However, we observed a significant decrease in their concentration in the study areas in the last two decades, related to an intensification of the pollen season. Because of the different effects air pollutants can have on the intensity of the pollen season and their interactions with other environmental parameters, further investigation should be carried out in this area.

## Conclusion

The pollen season of *Fraxinus*, *Quercus* and *Ambrosia* in Bratislava and B. Bystrica changed during the last two decades. The pollen season now starts earlier and is more intense, especially for trees, ends later for *Ambrosia* and lasts longer for *Quercus* and *Ambrosia*. Rising air temperature pre-season is responsible for the earlier beginning of tree pollen season (more significant for *Quercus*) and for its intensification, together with the influence of increased relative air humidity and/or precipitation. The combination of warm and humid weather conditions towards the end of summer and autumn enables an extended blooming period for *Ambrosia*. The relationship between air pollutant levels and the intensity of pollen season can be both positive (CO) and negative (SO_2_ and NO_2_), but their airborne concentration is also influenced by meteorological parameters, making the evaluation of their impact difficult.

These changes in the characteristics of pollen season of these allergenic tree and herbaceous taxa can harm the sensitive part of the population, especially in urban areas like the locations of our study. For the prognosis of the exacerbations of pollen-related allergenic diseases, it is necessary to further observe the development of these trends connected to the changing climate and atmospheric pollution.

### Supplementary Information

Below is the link to the electronic supplementary material.

Supplementary file1 (DOCX 46 KB)

## Data Availability

All data generated or analysed during this study are included in this published article.

## Abbreviations

MPS: Main pollen season
SHMÚ: Slovak Hydrometeorological Institute
HD: Number of high days
FHD: The first high day
SPIn: Seasonal pollen integral

## References

[CR1] Adams-Groom B, Selby K, Derrett S, Frisk CA, Pashley CH, Satchwell J, King D, McKenzie G, Neilson R (2022). Pollen season trends as markers of climate change impact: *Betula*, *Quercus* and Poaceae. Sci Total Environ.

[CR2] Anderegg WRL, Abatzoglou JT, Anderegg LDL, Ziska L (2021). Anthropogenic climate change is worsening North American pollen seasons. PNAS.

[CR3] Berger M, Bastl K, Bastl M, Dirr L, Hutter HP, Moshammer H, Gstöttner W (2020). Impact of air pollution on symptom severity during the birch, grass and ragweed pollen period in Vienna, Austria: importance of O_3_ in 2010–2018. Environ Pollut.

[CR4] Bogawski P, Grewling L, Nowak M, Smith M, Jackowiak B (2014). Trends in atmospheric concentrations of weed pollen in the context of recent climate warming in Poznań (Western Poland). Int J Biometeorol.

[CR5] Chen ZY, Petetin H, Méndez Turrubiates RF, Achebak H, García-Pando CP, Ballester J (2024). Population exposure to multiple air pollutants and its compound episodes in Europe. Nat Commun.

[CR6] Cheng X, Frank U, Zhao F, Capella JR, Winkler JB, Schnitzler J-P, Ghirardo A, Bertić M, Estrella N, Durner J, Pritsch K (2023). Plant growth traits and allergenic potential of *Ambrosia artemisiifolia* pollen as modified by temperature and NO_2_. Environ Exp Bot.

[CR7] Cunze S, Leiblein MC, Tackenberg O (2013) Range expansion of *Ambrosia artemisiifolia* in Europe is promoted by climate change. ISRN Ecol 2013:610126. 10.1155/2013/610126

[CR8] D’Amato G, Holgate ST, Pawankar R, Ledford DK, Cecchi L, Al-Ahmad (2015). Meteorological conditions, climate change, new emerging factors, and asthma and related allergic disorders. A statement of the World Allergy Organization. World Allergy Organ J.

[CR9] D’Amato G, Chong-Neto HJ, Monge Ortega OP, Vitale C, Ansotegui I, Rosario N, Haahtela T, Galan C, Pawankar R, Murrieta-Aguttes M, Cecchi L, Bergmann Ch, Ridolo E, Ramon G, Diaz SG, D’Amato M, Annesi-Maesano I (2020). The effects of climate change on respiratory allergy and asthma induced by pollen and mold allergens. Allergy.

[CR10] D’Amato G, Annesi-Maesano I, Biagioni B, Lancia A, Cecchi L, D’Ovidio MC, D’Amato M (2023). New developments in climate change, air pollution, pollen allergy, and interaction with SARS-CoV-2. Atmosphere.

[CR11] Damialis A, Halley JM, Giolekas D, Vokou D (2007). Long-term trends in atmospheric pollen levels in the city of Thessaloniki, Greece. Atmos Environ.

[CR12] de Weger LA, Bruffaerts N, Koenders MMJF, Verstraeten WW, Delcloo AW, Hentges P, Hentges F (2021). Long-term pollen monitoring in the Benelux: evaluation of allergenic pollen levels and temporal variations of pollen seasons. Front Allergy.

[CR13] El Kelish A, Zhao F, Heller W, Durner J, Winkler JB, Behrendt H, Traidl-Hoffmann C, Horres R, Pfeifer M, Frank U, Ernst D (2014). Ragweed (*Ambrosia artemisiifolia*) pollen allergenicity: SuperSAGE transcriptomic analysis upon elevated CO_2_ and drought stress. BMC Plant Biol.

[CR14] Frenz DA (2021). Interpreting atmospheric pollen counts for use in clinical allergy: allergic symptomology. Ann Allergy Asthma Immunol.

[CR15] Galán C, Cariñanos P, Alcázar P, Domínguez-Vilches E (2007). Spanish aerobiology network (REA): management and quality manual.

[CR16] Gehrig R, Clot B (2021). 50 years of pollen monitoring in Basel (Switzerland) demonstrate the influence of climate change on airborne pollen. Front Allergy.

[CR17] Gilles S, Akdis C, Lauener R, Schmid-Grendelmeier P, Bieber T, Schäppi G, Traidl-Hoffmann C (2018). The role of environmental factors in allergy: a critical reappraisal. Exp Dermatol.

[CR18] Glick S, Gehrig R, Eeftens M (2021). Multi-decade changes in pollen season onset, duration, and intensity: a concern for public health?. Sci Total Environ.

[CR19] Grewling Ł, Bogawski P, Kryza M, Magyar D, Šikoparija B, Skjøth CA, Udvardy O, Werner M, Smith M (2019). Concomitant occurrence of anthropogenic air pollutants, mineral dust and fungal spores during long-distance transport of ragweed pollen. Environ Pollut.

[CR20] Hamaoui-Laguel L, Vautard R, Liu L, Solmon F, Viovy N, Khvorostyanov D, Essl F, Chuine I, Colette A, Semenov MA, Schaffhauser A, Storkey J, Thibaudon M, Epstein MM (2015). Effects of climate change and seed dispersal on airborne ragweed pollen loads in Europe. Nat Clim Change.

[CR21] Hirst JM (1952). An automatic volumetric spore trap. Ann Appl Biol.

[CR22] Hrabovský M, Ščevková J, Mičieta K, Lafférsová J, Dušička J (2016). Expansion and aerobiology of *Ambrosia artemisiifolia* L. in Slovakia. Ann Agric Environ Med.

[CR23] Hrubiško M (1998). Pollinosis – an actual problem also in XXI. Century. Part III: Sequence and cross-reactivity of tree, grass and plant allergens by their clinical significance. Klin Imunológia a Alergol.

[CR24] Jochner S, Höfler J, Beck I, Göttlein A, Ankerst DP, Traidl-Hoffmann C, Menzel A (2013). Nutrient status: a missing factor in phenological and pollen research?. J Exp Bot.

[CR25] Jurko A (1990). Pollen allergens in our flora and vegetation. Naše Liečivé Rastliny.

[CR26] Levetin E (2021). Aeroallergens and climate change in Tulsa, Oklahoma: long-term trends in the south central United States. Frontiers in Allergy.

[CR27] Lind T, Ekebom A, Alm Kübler K, Östensson P, Bellander T, Lõhmus M (2016). Pollen season trends (1973–2013) in Stockholm Area, Sweden. Plos One.

[CR28] Makra L, Matyasovszky I, Tusnády G, Wang Y, Csépe Z, Bozóki Z (2016). Biogeographical estimates of allergenic pollen transport over regional scales: common ragweed and Szeged, Hungary as a test case. Agric For Meteorol.

[CR29] Oduber F, Calvo AI, Blanco-Alegre C, Castro A, Vega-Maray AM, Valencia-Barrera RM, Fernández-González D, Fraile R (2019). Links between recent trends in airborne pollen concentration, meteorological parameters and air pollutants. Agric for Meteorol.

[CR30] Peel RG, Ørby PV, Skjøth CA, Kennedy R, Schlünssen V, Smith M, Sommer J, Hertel O (2014). Seasonal variation in diurnal atmospheric grass pollen concentration profiles. Biogeosciences.

[CR31] Petrášová M, Jarolímek I (2012). Hardwood floodplain forests in Slovakia: syntaxonomical revision. Biologia.

[CR32] Rauer D, Gilles S, Wimmer M, Frank U, Mueller C, Musiol S, Vafadari B, Aglas L, Ferreira F, Kopplin-Schmitt P, Durner J, Winkler JB, Ernst D, Behrendt H, Weber-Schmidt CB, Hoffmann-Traidl C, Alessandrini F (2021). Ragweed plants grown under elevated CO_2_ levels produce pollen which elicit stronger allergic lung inflammation. Allergy.

[CR33] Reháčková T, Pauditšová E (2004). Evaluation of urban green spaces in Bratislava. Boreal Environ Res.

[CR34] Rodinkova V, Kremenska L, Palamarchuk O, Motruk I, Alexandrova E, Dudarenko O, Vakolyuk L, Yermishev O (2018). Seasonal changes in plant pollen concentrations over recent years in Vinnytsya, Central Ukraine. Acta Agrobot.

[CR35] Rogers CA, Wayne PM, Macklin EA, Muilenberg ML, Wagner CJ, Epstein PR, Bazzaz FA (2006). Interaction of the onset of spring and elevated atmospheric CO_2_ on ragweed (*Ambrosia artemisiifolia* L.) pollen production. Environ Health Perspect.

[CR36] Rojo J, Picornell A, Oteros J, Werchan M, Werchan B, Bergmann KC, Smith M, Weichenmeier I, Schmidt-Weber CB, Buters J (2021). Consequences of climate change on airborne pollen in Bavaria, Central Europe. Reg Environ Change.

[CR37] Ruiz-Valenzuela L, Aguilera F (2018). Trends in airborne pollen and pollen-season-related features of anemophilous species in Jaen (south Spain): a 23-year perspective. Atmos Environ.

[CR38] Ščevková J, Dušička J, Hrabovský M, Vašková Z (2021). Trends in pollen season characteristics of *Alnus*, Poaceae and *Artemisia* allergenic taxa in Bratislava, central Europe. Aerobiologia.

[CR39] Ščevková J, Dušička J, Zahradníková E, Sepšiová R, Kováč J, Vašková Z (2023). Impact of meteorological parameters and air pollutants on airborne concentration of *Betula* pollen and Bet v 1 allergen. Environ Sci Pollut Res.

[CR40] Schramm PJ, Brown CL, Saha S, Conlon KC, Manangan AP, Bell JE, Hess JJ (2021). A systematic review of the effects of temperature and precipitation on pollen concentrations and season timing, and implications for human health. Int J Biometeorol.

[CR41] Skjøth CA, Sun Y, Karrer G, Sikoparija B, Smith M, Schaffner U, Müller-Schärer H (2019). Predicting abundances of invasive ragweed across Europe using a “top-down” approach. Sci Total Environ.

[CR42] Stępalska D, Myszkowska D, Wołek J, Piotrowicz K, Obtułowicz K (2008). The influence of meteorological factors on *Ambrosia* pollen loads in Cracow, Poland, 1995–2006. Grana.

[CR43] Stępalska D, Myszkowska D, Piotrowicz K, Kluska K, Chłopek K, Grewling Ł, Lafférsová J, Majkowska-Wojciechowska B, Malkiewicz M, Piotrowska-Weryszko K, Puc M, Rodinkova V, Rybníček O, Ščevková J, Voloshchuk K (2020). High *Ambrosia* pollen concentrations in Poland respecting the long distance transport (LDT). Sci Total Environ.

[CR44] Takizawa H (2011). Impact of air pollution on allergic diseases. Korean J Intern Med.

[CR45] Theil H (1950). A rank-invariant method of linear and polynomial regression analysis, I. Proc K Ned Akad Wet.

[CR46] Tu W, Xiong Q, Qiu X, Zhang Y (2021). Dynamics of invasive alien plant species in China under climate change scenarios. Ecol Indic.

[CR47] Ulutaş K, Abujayyab SKM, Abu Amr SS (2021). Evaluation of the major air pollutants levels and its interactions with meteorological parameters in Ankara. J Eng Sci Des.

[CR48] Wopfner N, Gadermaier G, Egger M, Asero R, Ebner C, Jahn-Schmid B, Ferreira F (2005). The spectrum of allergens in ragweed and mugwort pollen. Int Arch Allergy Immunol.

[CR49] Zhang Y, Bielory L, Mi Z, Cai T, Robock A, Georgopoulos P (2015). Allergenic pollen season variations in the past two decades under changing climate in the United States. Glob Change Biol.

[CR50] Zhao F, Heller W, Stich S, Durner J, Winkler JB, Traidl-Hoffmann C, Ernst D, Frank U (2017). Effects of NO_2_ on inflorescence length, pollen/seed amount and phenolic metabolites of common ragweed (*Ambrosia artemisiifolia* L.). Am J Polit Sci.

[CR51] Zhao W, Xue Z, Liu T, Wang H, Han Z (2023). Factors affecting establishment and population growth of the invasive weed *Ambrosia artemisiifolia*. Front Plant Sci.

[CR52] Ziska LH (2021). Climate, carbon dioxide, and plant-based aero-allergens: a deeper botanical perspective. Front Allergy.

[CR53] Ziska LH, Caulfield FA (2000). Rising CO_2_ and pollen production of common ragweed (*Ambrosia artemisiifolia*), a known allergy-inducing species: implications for public health. Aust J Plant Physiol.

[CR54] Ziska L, Makra L, Harry S, Bruffaerts N, Hendrickx M, Coates F, Saarto A, Thibaudon M, Oliver G, Damialis A, Charalampopoulos A, Vokou D, Heiðmarsson S, Guđjohnsen E, Bonini M, Oh J-W, Sullivan K, Ford L, Brooks G, Crimmins A (2019). Temperature-related changes in airborne allergenic pollen abundance and seasonality across the northern hemisphere: a retrospective data analysis. Lancet Planet Health.

