# Supplementary material for: Long-term pollen season trends of Fraxinus (ash), Quercus (oak) and Ambrosia artemisiifolia (ragweed) as indicators of anthropogenic climate change impact
Source: Environ Sci Pollut Res Int. 2024 Jun 19;31(30):43238–48. doi: 10.1007/s11356-024-34027-w (PMC11222177; doi:10.1007/s11356-024-34027-w)
Supplement: Supplementary file 1 — Supplementary file1 (DOCX 46 KB) [file 11356_2024_34027_MOESM1_ESM.docx]

**Long-term pollen season trends of *Fraxinus* (ash), *Quercus* (oak) and *Ambrosia* *artemisiifolia* (ragweed) as indicators of anthropogenic climate change impact**

^1^Jana Ščevková, ^1^Natália Štefániková, ^1^Jozef Dušička, ^2^Janka Lafférsová, ^1^Eva Zahradníková

**SUPPLEMENTARY INFORMATION:**

**Table S1.** *Fraxinus* pollen-season-related characteristics

| Year | Area | Pollen-season-related characteristics | | | | | | |
| --- | --- | --- | --- | --- | --- | --- | --- | --- |
|  |  | SD | ED | D | PV | HD | FHD | SPIn |
| 2002 | Ba | 64 | 120 | 57 | 384 | 17 | 65 | 2,971 |
|  | BB | 77 | 125 | 49 | 43 | 3 | 78 | 578 |
| 2003 | Ba | 85 | 124 | 40 | 496 | 17 | 87 | 3,154 |
|  | BB | 89 | 140 | 52 | 64 | 3 | 112 | 526 |
| 2004 | Ba | 79 | 119 | 41 | 175 | 7 | 80 | 1,078 |
|  | BB | 97 | 120 | 24 | 30 | 0 | 105 | 266 |
| 2005 | Ba | 88 | 121 | 34 | 223 | 4 | 88 | 954 |
|  | BB | 100 | 127 | 28 | 63 | 3 | 116 | 399 |
| 2006 | Ba | 97 | 117 | 21 | 413 | 12 | 100 | 1,805 |
|  | BB | 113 | 133 | 21 | 105 | 1 | 113 | 246 |
| 2007 | Ba | 75 | 106 | 32 | 56 | 1 | 91 | 472 |
|  | BB | 85 | 120 | 36 | 22 | 0 | 105 | 234 |
| 2008 | Ba | 61 | 96 | 36 | 37 | 0 | 83 | 353 |
|  | BB | 77 | 129 | 53 | 138 | 5 | 101 | 653 |
| 2009 | Ba | 93 | 99 | 7 | 42 | 0 | 95 | 108 |
|  | BB | 89 | 122 | 34 | 67 | 5 | 105 | 539 |
| 2010 | Ba | 85 | 111 | 27 | 22 | 0 | 110 | 217 |
|  | BB | 86 | 136 | 51 | 183 | 7 | 118 | 802 |
| 2011 | Ba | 83 | 113 | 31 | 160 | 6 | 89 | 916 |
|  | BB | 90 | 145 | 56 | 50 | 1 | 122 | 489 |
| 2012 | Ba | 79 | 109 | 31 | 180 | 10 | 84 | 1,272 |
|  | BB | 76 | 144 | 69 | 161 | 20 | 95 | 1,828 |
| 2013 | Ba | 98 | 121 | 24 | 216 | 8 | 105 | 1,344 |
|  | BB | 94 | 160 | 67 | 600 | 16 | 108 | 4,357 |
| 2014 | Ba | 58 | 99 | 42 | 62 | 3 | 74 | 1,081 |
|  | BB | 76 | 124 | 49 | 58 | 12 | 81 | 1,092 |
| 2015 | Ba | 67 | 114 | 48 | 296 | 15 | 82 | 2,368 |
|  | BB | 84 | 131 | 48 | 255 | 24 | 100 | 2,463 |
| 2016 | Ba | 53 | 108 | 56 | 126 | 10 | 62 | 1,468 |
|  | BB | 90 | 150 | 61 | 40 | 2 | 108 | 503 |
| 2017 | Ba | 72 | 105 | 34 | 298 | 20 | 74 | 2,887 |
|  | BB | 81 | 132 | 52 | 573 | 20 | 83 | 2,808 |
| 2018 | Ba | 88 | 113 | 26 | 1,448 | 7 | 98 | 3,250 |
|  | BB | 97 | 130 | 34 | 130 | 19 | 102 | 1,639 |
| 2019 | Ba | 65 | 109 | 45 | 112 | 7 | 67 | 1,180 |
|  | BB | 81 | 121 | 41 | 75 | 8 | 89 | 815 |
| 2020 | Ba | 47 | 116 | 70 | 1,182 | 36 | 54 | 8,063 |
|  | BB | 70 | 140 | 71 | 246 | 25 | 79 | 2,364 |
| 2021 | Ba | 56 | 121 | 66 | 585 | 15 | 70 | 3,166 |
|  | BB | 84 | 132 | 49 | 56 | 3 | 84 | 479 |
| 2022 | Ba | 49 | 108 | 60 | 1,360 | 35 | 54 | 9,143 |
|  | BB | - | - | - | - | - | - | - |
| 2002‒2022 | Ba | 73 | 112 | 39 | 375 | 11 | 82 | 2,250 |
| 2002‒2021 | BB | 87 | 133 | 47 | 148 | 9 | 100 | 1,154 |

*Ba –* Bratislava; *BB* – B. Bystrica; *SD* – start date (DOY); *ED* – end date (DOY); *D* – duration (days); *PV* – peak value (pollen/m^3^); *HD* – high days (number); *FHD* – first high day (DOY); *SPIn* – seasonal pollen integral (pollen*day/m^3^)

**Table S2.** *Quercus* pollen-season-related characteristics

| Year | Area | Pollen-season-related characteristics | | | | | | |
| --- | --- | --- | --- | --- | --- | --- | --- | --- |
|  |  | SD | ED | D | PV | HD | FHD | SPIn |
| 2002 | Ba | 81 | 129 | 49 | 151 | 8 | 117 | 978 |
|  | BB | 119 | 135 | 17 | 38 | 2 | 125 | 241 |
| 2003 | Ba | 114 | 129 | 16 | 311 | 11 | 116 | 1,784 |
|  | BB | 119 | 142 | 24 | 302 | 8 | 120 | 1,022 |
| 2004 | Ba | 118 | 141 | 24 | 25 | 0 | 120 | 168 |
|  | BB | 112 | 132 | 21 | 34 | 1 | 123 | 202 |
| 2005 | Ba | 97 | 126 | 30 | 174 | 5 | 98 | 664 |
|  | BB | 114 | 145 | 32 | 22 | 0 | 126 | 208 |
| 2006 | Ba | 115 | 131 | 17 | 136 | 4 | 116 | 559 |
|  | BB | 120 | 144 | 25 | 76 | 3 | 124 | 388 |
| 2007 | Ba | 107 | 121 | 15 | 35 | 0 | 116 | 184 |
|  | BB | 113 | 131 | 19 | 34 | 1 | 119 | 197 |
| 2008 | Ba | 114 | 122 | 9 | 27 | 0 | 117 | 133 |
|  | BB | 117 | 149 | 33 | 52 | 2 | 137 | 283 |
| 2009 | Ba | 105 | 121 | 17 | 61 | 2 | 118 | 375 |
|  | BB | 96 | 130 | 35 | 247 | 11 | 107 | 1,813 |
| 2010 | Ba | 110 | 126 | 17 | 100 | 4 | 114 | 571 |
|  | BB | 116 | 142 | 27 | 101 | 8 | 119 | 671 |
| 2011 | Ba | 107 | 125 | 19 | 201 | 6 | 109 | 1,066 |
|  | BB | 94 | 146 | 53 | 312 | 12 | 111 | 2,379 |
| 2012 | Ba | 94 | 127 | 34 | 72 | 4 | 119 | 676 |
|  | BB | 95 | 133 | 39 | 224 | 14 | 112 | 1,637 |
| 2013 | Ba | 106 | 129 | 24 | 105 | 1 | 121 | 546 |
|  | BB | 112 | 142 | 31 | 538 | 16 | 113 | 3,027 |
| 2014 | Ba | 89 | 124 | 36 | 131 | 5 | 96 | 1,157 |
|  | BB | 80 | 142 | 63 | 46 | 5 | 81 | 750 |
| 2015 | Ba | 110 | 132 | 23 | 47 | 0 | 113 | 465 |
|  | BB | 100 | 145 | 46 | 396 | 16 | 103 | 2,085 |
| 2016 | Ba | 104 | 140 | 37 | 291 | 12 | 104 | 2,024 |
|  | BB | 106 | 150 | 45 | 97 | 10 | 106 | 839 |
| 2017 | Ba | 92 | 138 | 47 | 390 | 20 | 100 | 3,321 |
|  | BB | 83 | 147 | 65 | 255 | 17 | 91 | 1,760 |
| 2018 | Ba | 106 | 127 | 22 | 497 | 9 | 107 | 2,673 |
|  | BB | 107 | 139 | 33 | 384 | 14 | 110 | 2,197 |
| 2019 | Ba | 98 | 128 | 31 | 507 | 16 | 99 | 2,897 |
|  | BB | 91 | 129 | 39 | 211 | 4 | 111 | 706 |
| 2020 | Ba | 77 | 103 | 27 | 138 | 5 | 78 | 836 |
|  | BB | 70 | 155 | 86 | 304 | 35 | 70 | 4,040 |
| 2021 | Ba | 90 | 144 | 55 | 358 | 11 | 120 | 2,055 |
|  | BB | 95 | 160 | 66 | 113 | 8 | 111 | 786 |
| 2022 | Ba | 84 | 142 | 59 | 1,158 | 22 | 106 | 6,808 |
|  | BB | - | - | - | - | - | - | - |
| 2002‒2022 | Ba | 101 | 129 | 29 | 234 | 7 | 110 | 1,426 |
| 2002‒2021 | BB | 103 | 142 | 40 | 189 | 9 | 111 | 1,262 |

*Ba –* Bratislava; *BB* – B. Bystrica; *SD* – start date (DOY); *ED* – end date (DOY); *D* – duration (days); *PV* – peak value (pollen/m^3^); *HD* – high days (number); *FHD* – first high day (DOY); *SPIn* – seasonal pollen integral (pollen*day/m^3^)

**Table S3.** *Ambrosia* pollen-season-related characteristics

| Year | Area | Pollen-season-related characteristics | | | | | | |
| --- | --- | --- | --- | --- | --- | --- | --- | --- |
|  |  | SD | ED | D | PV | HD | FHD | SPIn |
| 2002 | Ba | 222 | 254 | 33 | 342 | 24 | 222 | 2,883 |
|  | BB | 236 | 258 | 23 | 153 | 8 | 239 | 392 |
| 2003 | Ba | 229 | 271 | 43 | 216 | 14 | 230 | 1,360 |
|  | BB | 230 | 264 | 35 | 76 | 6 | 243 | 259 |
| 2004 | Ba | 224 | 266 | 43 | 174 | 22 | 225 | 1,703 |
|  | BB | 228 | 258 | 31 | 198 | 11 | 228 | 907 |
| 2005 | Ba | 225 | 270 | 46 | 332 | 20 | 225 | 2,028 |
|  | BB | 236 | 257 | 22 | 66 | 3 | 253 | 185 |
| 2006 | Ba | 228 | 269 | 42 | 243 | 19 | 230 | 2,187 |
|  | BB | 229 | 261 | 33 | 18 | 4 | 268 | 151 |
| 2007 | Ba | 227 | 268 | 42 | 156 | 18 | 227 | 1,143 |
|  | BB | 229 | 243 | 15 | 26 | 6 | 236 | 152 |
| 2008 | Ba | 225 | 263 | 39 | 57 | 17 | 232 | 862 |
|  | BB | 220 | 257 | 38 | 204 | 16 | 242 | 1,319 |
| 2009 | Ba | 222 | 262 | 41 | 57 | 20 | 229 | 954 |
|  | BB | 214 | 259 | 46 | 536 | 23 | 240 | 2,584 |
| 2010 | Ba | 223 | 268 | 46 | 37 | 8 | 233 | 659 |
|  | BB | 201 | 237 | 37 | 282 | 6 | 234 | 727 |
| 2011 | Ba | 219 | 268 | 50 | 149 | 30 | 225 | 2,007 |
|  | BB | 202 | 272 | 71 | 266 | 34 | 239 | 2,523 |
| 2012 | Ba | 227 | 270 | 44 | 120 | 27 | 230 | 1,627 |
|  | BB | 219 | 280 | 62 | 123 | 24 | 250 | 1,175 |
| 2013 | Ba | 219 | 263 | 45 | 290 | 28 | 229 | 1,949 |
|  | BB | 214 | 269 | 56 | 37 | 9 | 241 | 356 |
| 2014 | Ba | 215 | 263 | 49 | 92 | 34 | 223 | 1,585 |
|  | BB | 213 | 268 | 56 | 163 | 15 | 259 | 869 |
| 2015 | Ba | 221 | 268 | 48 | 230 | 25 | 225 | 1,822 |
|  | BB | 218 | 284 | 67 | 367 | 22 | 242 | 2,014 |
| 2016 | Ba | 217 | 263 | 47 | 274 | 27 | 230 | 2,406 |
|  | BB | 217 | 260 | 44 | 162 | 20 | 263 | 892 |
| 2017 | Ba | 220 | 272 | 53 | 414 | 17 | 227 | 1,848 |
|  | BB | 213 | 268 | 56 | 181 | 17 | 259 | 1,549 |
| 2018 | Ba | 200 | 286 | 87 | 246 | 41 | 210 | 2,479 |
|  | BB | 220 | 301 | 82 | 185 | 25 | 277 | 1,301 |
| 2019 | Ba | 225 | 266 | 42 | 203 | 25 | 225 | 1,770 |
|  | BB | 224 | 275 | 52 | 267 | 17 | 264 | 1,247 |
| 2020 | Ba | 226 | 277 | 52 | 315 | 22 | 226 | 2,038 |
|  | BB | 220 | 278 | 59 | 303 | 17 | 256 | 1,327 |
| 2021 | Ba | 225 | 278 | 54 | 375 | 19 | 234 | 1,976 |
|  | BB | 219 | 278 | 60 | 109 | 11 | 272 | 623 |
| 2022 | Ba | 218 | 278 | 61 | 256 | 21 | 225 | 2,077 |
|  | BB | - | - | - | - | - | - | - |
| 2002‒2022 | Ba | 222 | 269 | 48 | 218 | 23 | 227 | 1,779 |
| 2002‒2021 | BB | 220 | 266 | 47 | 186 | 15 | 250 | 1,028 |

*Ba –* Bratislava; *BB* – B. Bystrica; *SD* – start date (DOY); *ED* – end date (DOY); *D* – duration (days); *PV* – peak value (pollen/m^3^); *HD* – high days (number); *FHD* – first high day (DOY); *SPIn* – seasonal pollen integral (pollen*day/m^3^)
